# The Role of Promyelocytic Leukemia Nuclear Bodies During HPV Infection

**DOI:** 10.3389/fcimb.2020.00035

**Published:** 2020-02-19

**Authors:** Lucile G. Guion, Martin Sapp

**Affiliations:** ^1^Department of Microbiology and Immunology, Center for Molecular and Tumor Virology, Louisiana State University Health Sciences Center, Shreveport, LA, United States; ^2^Feist Weiller Cancer Center, Louisiana State University Health Sciences Center, Shreveport, LA, United States

**Keywords:** PML, HPV—human papillomavirus, infectious entry, PML nuclear bodies, L2 protein, Innate immnuity, transcription

## Abstract

Promyelocytic leukemia (PML) nuclear bodies (NBs) are highly dynamic subnuclear structures. Their name giving major component, PML protein, is essential for their formation. PML is present in many different isoforms due to differential splicing, which seem to contribute differently to PML NBs function. Sp100 and DAXX are also permanently residing in these structures. PML NBs disassemble in mitosis to form large cytoplasmic aggregates and reassemble after completion of cell division. Posttranslational modifications such as SUMOylation play important roles for protein association with PML NBs. In addition to the factors permanently associated with PML NBs, a large number of proteins may transiently reside in PML NBs dependent on cell stage, type, and condition. PML NBs have been indirectly implicated in a large number of cellular processes including apoptosis, transcriptional regulation, DNA repair and replication. They are considered hot spots for posttranslational modifications and may serve as readily accessible protein depots. However, a precise function has been difficult to assign. Many DNA viruses target PML NBs after entry often resulting in reorganization of these subnuclear structures. Antiviral activity has been assigned to PML NBs partially based on the observation that PML protein is an interferon stimulated gene. In contrast, human papillomavirus (HPV) infection requires the presence of PML protein suggesting that PML NBs may be essential to establish infection. This review will summarize and discuss recent advances in our understanding of the role of PML NBs and individual protein components in the establishment of HPV infection.

## Introduction

In this review, we are discussing the role promyelocytic leukemia (PML) nuclear bodies (NBs) play during infection with human papillomavirus (HPV). We will compare with how other viruses interact with PML NBs and the outcome for establishment of infection. The function of PML NBs has been difficult to pinpoint due to their association with many cellular processes. With their insoluble nature, PML NBs cannot be pulled down and studying these structures has been challenging, also contributing to their poor understanding. New microscopy techniques, particularly high-resolution microscopy and three-dimensional reconstruction of images, have greatly advanced our way of studying PML NBs in recent years, and thus have led to better understanding of their role in the context of viral infection.

## Promyelocytic Leukemia Nuclear Bodies

PML NBs, also known as nuclear domain 10 or promyelocytic oncogenic domains, are punctate ring-shaped dynamic structures present in the nucleus of most cell types. They have been associated with numerous cellular processes, such as cell cycle, apoptosis, stress, transcriptional regulation, genome stability, tumor suppression, and innate and intrinsic immune responses (Bernardi and Pandolfi, 2007). PML NBs consist of many permanently and transiently associated proteins, such as transcription factors, oncogenes, and enzymes involved in post-translational protein modifications, although the number and variety of residing proteins can vary greatly depending on the cell condition. Due to the diverse nature of residing proteins and cellular processes with which PML NBs are associated, the distinct biochemical functions of PML NBs has been difficult to specifically identify. These structures have been proposed to be nuclear depots for protein storage, sites of nuclear activities, specifically transcriptional activation and repression, involved in DNA replication and repair, posttranscriptional regulation of gene expression, posttranslational modifications, and/or intrinsic immune response (Borden et al., 1995; Scherer and Stamminger, 2016).

PML NBs are thought to be hotspots for translational modifications since nearly all PML NB-residing proteins are modified by small ubiquitin-like modifiers (SUMOs) (Pichler et al., 2017). SUMOs are ubiquitin-related peptides present in three isoforms (SUMO 1-3) that are reversibly and dynamically conjugated to target proteins on lysine residues within a specific consensus sequence (ΨKxE). The mechanism of SUMO conjugation is similar to ubiquitylation, as it involves an E1 activating enzyme, an E2 conjugating enzyme, and an E3 ligase. SUMOylation has been shown to regulate protein stability, subcellular localization, and protein-protein interaction. Indeed, SUMO can promote non-covalent binding to other proteins harboring a specific motif that contains a hydrophobic core followed by an acidic region, called a SUMO interacting motif (SIM) (Minty et al., 2000; Hecker et al., 2006). SUMO conjugation and interaction are required for the formation, stability, and localization of PML NBs, as well as the ability to interact with and recruit other PML NB-associated proteins (Sternsdorf et al., 1997a; Zhong et al., 2000; Shen et al., 2006). Also, stress induced PML NB assembly is controlled by SUMOylation of partner proteins (Sahin et al., 2014).

## PML Protein

The major component of PML NBs is the tumor suppressor PML protein, which is critical for the formation and stability of these structures (Ishov et al., 1999). The importance of PML protein was first reported in acute promyelocytic leukemia, a rare but extremely malignant condition due to the abnormal fusion of PML protein with the retinoic acid receptor α. The fusion protein blocks transcription and granulocyte differentiation, leading to the accumulation of immature granulocytes (Daniel et al., 1993; de The et al., 2012). PML protein harbors a RING-finger, two B-boxes, and an α-helical coiled coil domain together forming an RBCC/TRIM motif in exons 1-3 (Nisole et al., 2013). PML protein is present in seven isoforms through alternative splicing. PML I-VI localize mainly in the nucleus to form PML NBs, whereas PML VII lacks a nuclear localization signal and therefore remains in the cytosol. PML protein forms the structural scaffold of PML NBs as an outer shell, which is essential as cells knocked down for PML protein fail to form these structures (Ishov et al., 1999; Zhong et al., 2000). The assembly of PML proteins into PML NBs also depends on their SUMOylation (Zhong et al., 2000; Shen et al., 2006). PML protein contains four SUMO conjugation motifs (K65, K160, K490, K616) and one SIM, allowing for interaction with other SUMOylated PML proteins (Sternsdorf et al., 1997b; Kamitani et al., 1998; Cuchet-Lourenco et al., 2011). Following PML protein SUMOylation, SUMO conjugation motifs and the RBCC motif promote the nucleation of the PML structure through non-covalent interactions between PML molecules (Shen et al., 2006). Subsequently, other PML NB-associated proteins are recruited through SUMOylation and binding to SIMs on PML NB-associated proteins.

## SP100

The transcriptional repressor Sp100 also permanently resides within PML NBs (Sternsdorf et al., 1995). Sp100 is an acidic protein of approximately 53 kDa that harbors an aberrant electrophoretic mobility in SDS-polyacrylamide gels of 100 kDa (Szostecki et al., 1987). Sp100 is present in four isoforms as a result of alternative splicing, Sp100A, B, C, and HMG (Seeler et al., 2001). All isoforms but Sp100A contain a SAND DNA-binding domain. The SAND domain is found in the protein family of Sp100, AIRE-1, NucP41/75, and DEAF-1 and is involved in chromatin-dependent transcriptional regulation. Interestingly, while most Sp100 isoforms are known transcriptional repressors of viruses, Sp100A enhances HSV-1 gene expression (Negorev et al., 2006). Sp100 can be SUMOylated, whereupon it can interact with PML protein and associate with PML NBs (Sternsdorf et al., 1997b). However, the role of SUMO modification for Sp100 is not entirely understood, as SUMOylation may not be essential for Sp100 targeting to PML NBs (Sternsdorf et al., 1999). Indeed, unSUMOylated Sp100 were found to target PML NBs, possibly through SUMO interactions between a SUMO on PML protein and a SIM on Sp100.

## Viruses Interact With PML NBs to Establish Infection

Many viruses, specifically nuclear-replicating viruses, have been shown to target PML NBs as a critical step for establishing productive infection (Ishov and Maul, 1996). Simian virus 40 (SV40), adenoviruses (ADV), human cytomegalovirus (HCMV), and HSV-1 all induce the reorganization of PML NB-residing proteins, resulting in the disassembly and/or degradation of the structures, which is required to initiate viral transcription and replication (Everett and Maul, 1994; Ishov and Maul, 1996; Everett et al., 1998; Kim et al., 2011; Berscheminski et al., 2013). Indeed, PML NBs have been implicated in intrinsic immunity, the first intracellular response against invading pathogens, and are thought to act as sensors of DNA/protein complexes (Rivera-Molina et al., 2012; Scherer and Stamminger, 2016). Viral infection has been used extensively to study the roles of PML NBs in innate immune signaling as repressors of viral gene expression and coregulators of interferon (IFN) responses. PML protein is most notably known to contribute to an interferon (IFN)-induced antiviral state in HSV-1 and influenza infections (Regad et al., 2001; Chee et al., 2003). However, the precise molecular mechanisms by which PML protein stimulates IFN pathways are still poorly understood. PML protein is thought to associate with transcription factors, such as STAT1 and IRF3, which are involved in regulating IFN and IFN-stimulated gene (ISG) expression (Chen et al., 2015). Furthermore, PML NB-associated transcriptional repressors, such as Sp100 and DAXX, have been shown to restrict viral gene expression in HSV-1, ADV, HPV, Epstein Barr Virus (EBV), and HCMV infection (Negorev et al., 2006; Kim et al., 2011; Schreiner et al., 2013; Stepp et al., 2013; Tsai et al., 2014; Albright and Kalejta, 2016).

To overcome the antiviral environment induced by PML NBs, viruses have evolved effector proteins to serve as antagonists (Scherer and Stamminger, 2016). In most cases, these viral proteins are products of immediate early genes, due to their critical role in establishment of infection. ADV immediate early protein E1A-13S has been shown to specifically target PML II for degradation, which enhances viral gene expression (Berscheminski et al., 2013). HCMV targets Sp100 for degradation through immediate early protein IE1 to prevent gene expression silencing (Kim et al., 2011). However, tegument proteins, for example HCMV pp71, have also been shown to directly target PML NB components such as DAXX (Saffert and Kalejta, 2006; Hwang and Kalejta, 2009).

The best studied interaction of a viral protein with PML NBs is HSV-1 immediate early protein ICP0. ICP0 localizes to PML NBs at early times of viral infection and was shown to disrupt and reorganize PML NBs, mainly by mediating the degradation of specific PML isoforms (Everett and Maul, 1994; Cuchet-Lourenco et al., 2012). ICP0 is also thought to be involved in the degradation of Sp100, as knockdown of Sp100 results in increased expression of ICP0-null mutant HSV-1 (Chelbi-Alix and de The, 1999; Negorev et al., 2006; Everett et al., 2008). Due to their possible role as sensors of DNA/protein complexes, PML NBs were shown to be recruited to virally-induced foci, likely in order to associate with HSV-1 genomes and repress gene expression (Everett et al., 2007; Alandijany et al., 2018). The association with PML NBs and especially DAXX in association with the histone H3.3 chaperone ATXR was shown to be essential for the induction of HSV-1 latency in neurons (Catez et al., 2012; Cohen et al., 2018). This recruitment is thought to be mediated by SUMO interactions or sensing of SUMO-1 conjugation motif on PML proteins by ICP0 (Boutell et al., 2003). The degradation of PML isoforms promoted by ICP0 was also shown to be SUMO-dependent (Boutell et al., 2011). Finally, recent high-resolution microscopy revealed that PML NB-associated proteins can engulf HSV-1 genomes shortly after nuclear entry (Alandijany et al., 2018).

## HPV Is Delivered Into the Nucleus in a Transport Vesicle

Incoming HPV target PML NBs after infectious entry into target keratinocytes. HPV are epitheliotropic viruses whose replication cycle is strictly dependent on the terminal differentiation process of keratinocytes of the skin and mucosa (Doorbar et al., 2015). To achieve entry, HPV preferentially binds to components of the basement membrane, which separates the epidermal from the dermal tissue (Culp et al., 2006a; Selinka et al., 2007; Richards et al., 2014). Animal models have revealed that access to the basement membrane requires disruption of the epidermal architecture such as wounds, abrasions or microlesions (Roberts et al., 2007). This seems to allow the virus to specifically target basal cells, the only dividing cells in this otherwise terminally differentiated tissue. Interaction with attachment receptors such as laminin 332 and heparan sulfate proteoglycans (Culp et al., 2006b; Selinka et al., 2007; Richards et al., 2013) triggers conformational changes and subsequent proteolytic processing of the major and minor capsid protein, L1 and L2, respectively. Conformational changes as well as proteolytic cleavage are mediated by host cell factors such as Cyclophilin B, furin convertases, and kallikrein 8 (Richards et al., 2006; Bienkowska-Haba et al., 2009; Cerqueira et al., 2015). Following transfer to uptake receptors likely involving tetraspanins and tetraspanin enriched microdomains, HPV are internalized by an endocytic event (Schelhaas et al., 2012; Spoden et al., 2013). Acidification of the endocytic vesicle induces partial uncoating, which triggers insertion of the L2 protein into the endocytic membrane resulting in a transmembranous configuration of L2 (Selinka et al., 2002; DiGiuseppe et al., 2015). This process is likely assisted by a previously unknown chaperone activity of γ-secretase (Karanam et al., 2013; Inoue et al., 2018). A short amino terminal peptide remains luminal (residues 13 to 44) and is separated by a transmembrane domain from the carboxyl terminus (residues 66 to 473), which is exposed to the cytosol. The carboxyl terminus of L2 harbors domains shown to interact with cytosolic transport factors such as retromer complexes that direct the transport of HPV-harboring vesicles to the trans golgi network (TGN) (Florin et al., 2006; Bergant Marusic et al., 2011; Schneider et al., 2011; Bergant and Banks, 2013; Lipovsky et al., 2013; Zhang et al., 2014, 2018). HPV requires mitosis and breakdown of the nuclear envelope for delivery into the nucleus (Pyeon et al., 2009; Aydin et al., 2014). Using differential permeabilization, differential staining, DNAse protection, and flotation assays, we recently demonstrated that the viral genome along with the major capsid protein L1 reside within an unidentified vesicular compartment (DiGiuseppe et al., 2016, 2017). This transmembranous configuration of L2 is retained during mitosis, probably mediating transport along mitotic microtubules toward condensed chromosomes (DiGiuseppe et al., 2016; Aydin et al., 2017; Day et al., 2019). The viral genome remains in the transport vesicle for a short time after completion of mitosis and nuclear envelope reformation (DiGiuseppe et al., 2016; Day et al., 2019).

## The Study of HPV Interacting With PML NBs

Similarly to other DNA viruses, HPV has been shown to interact with PML NBs after nuclear delivery as part of the life cycle. BPV 1 L2 protein was first found to localize to PML NBs when overexpressed in cell culture (Day et al., 1998). Overexpression of HPV 33 L2 protein also results in its targeting to PML NBs, as well as the displacement of Sp100 but not PML protein (Florin et al., 2002; Becker et al., 2003). This reorganization of PML NBs was also transiently observed in natural productive lesions (Florin et al., 2002). Later, HPV 16 L2 protein and BPV 1 pseudogenomes were found to localize at PML NBs (Day et al., 2004). Interestingly, L2 protein harbors a diffuse nuclear distribution in cells lacking PML protein.

## The Role of PML Protein for Establishment of HPV Infection

In order to better study the interactions of HPV particles and PML NBs and their outcomes for infection, pseudoviral and quasiviral vectors have recently been more widely used over overexpression systems. Quasivirions are generated by co-transfecting three expression plasmids that encode codon-optimized L1 and L2 genes, the HPV 16 genome flanked by LoxP sites on a GFP reporter, and Cre recombinase yielding virions encapsidating either the HPV16 genome to form quasivirions or the GFP-containing plasmid to form pseudovirions (PsVs) (Lee et al., 2004; Buck et al., 2005; Buck and Thompson, 2007). PsVs are generated by co-transfecting the L1/L2 expression plasmid and a GFP-expressing plasmid only (Unckell et al., 1997; Leder et al., 2001; Buck et al., 2004; Buck and Thompson, 2007). The pseudogenomes are labeled with 5-ethynyl-2′-deoxyuridine (EdU) during PsV generation for detection by fluorescent microscopy after the Click-iT reaction (Ishii et al., 2010).

PsVs were shown to be more efficient at infecting PML protein-expressing cells than PML knockdown cells (Day et al., 2004). In addition, BPV 1 transcript and genome levels are greatly diminished in cells lacking PML protein. This was the first evidence of the requirement of PML protein to enhance viral infectivity in the early stages of the PV life cycle. Furthermore, this study suggested that PML protein may provide an environment that favors viral transcription in the case of PV infection.

A previous study revealed that HPV 16 pseudogenomes colocalized with PML protein in both HaCaT and HeLa cells (Bienkowska-Haba et al.,). Although a significant decrease in viral transcripts in HaCaT cells knocked down for PML protein was observed, no differences in PML knocked down HeLa cells was apparent. Additionally, the number of pseudogenomes retained in the nucleus of HaCaT cells was greatly decreased in the absence of PML protein after successful nuclear delivery, whereas no difference was observed in HeLa cells in the presence or absence of PML protein. Loss of viral genomes in PML-deficient HaCaT cells was rescued by inhibiting the Jak/Stat signaling pathway, suggesting that immune signaling may be involved in the absence of PML protein. However, viral transcription was not restored. Taken together, the results suggested that PML protein may provide a protective environment for the viral genome against innate immune recognition and subsequent degradation. However, this is most likely not necessary in HeLa cells, which are already transformed with HPV 18 and therefore lack IFN response. Furthermore, it is tempting to assume that this protection of the viral genome by PML protein then results in efficient viral transcription and establishment of infection.

PML NBs are subject to modifications during cell cycle progression (Everett et al., 1999; Dellaire et al., 2006). Upon the onset of mitosis, PML protein is de-SUMOylated and Sp100 and other PML NB components dissociate from the PML protein structure. PML proteins then homo-multimerize through the RBCC/TRIM motif and form large aggregates in the cytosol. These mitotic accumulations of PML proteins (MAPPs) do not contain any other PML NB-associated protein and remain in the cytosol even after completion of mitosis and nuclear envelope reformation. Sp100 becomes diffuse in the cytosol during mitosis. PML protein is then recycled and translocates back into the nucleus of daughter cells, while new PML protein is also synthesized. Subsequently, PML protein is modified by SUMO to assemble into new PML NBs and recruit Sp100, DAXX and other PML NB-associated proteins.

**Figure 1:**
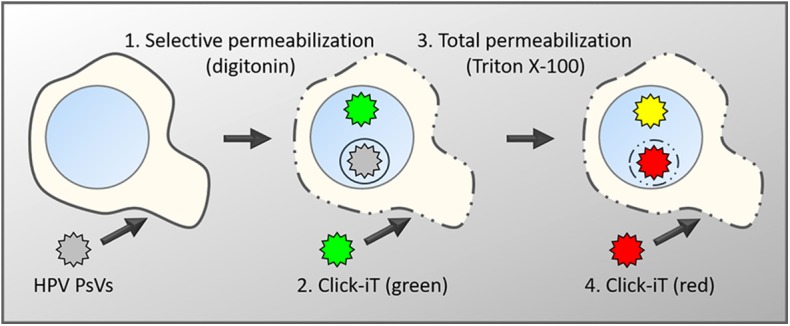
Schematic diagram of the differential staining technique. Keratinocytes are infected with PsVs, whose encapsidated DNA had been labeled with EdU during virus production (Ishii et al., 2010). After fixation, cells are differentially permeabilized with low concentrations of digitonin under conditions that will exclusively permeabilize membranes rich in cholesterol such as the plasma membrane and endocytic vesicles directly derived from the plasma membrane (1) (DiGiuseppe et al., 2016). Subsequently, accessible viral genome will be labeled with a green fluorescent dye using Click-iT chemistry (2). This is followed by a total permeabilization using high concentrations of Triton X-100 (3) and a second round of Click-iT reaction using a red fluorescent dye (4). Genomes accessible after the initial digitonin permeabilization will be labeled with both dyes and appear in yellow in fluorescent microscopy. Genomes residing in the lumen of intracellular membranes such as the TGN or transport vesicles will only be accessible to the red dye and therefore appear in red.

**Figure 2:**
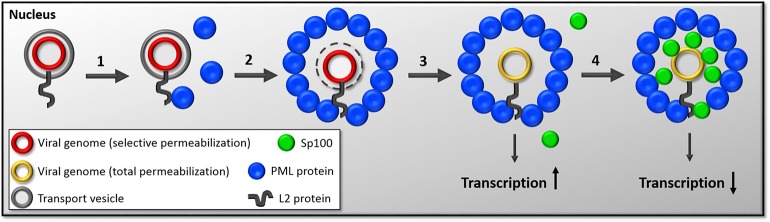
Diagram of the timeline of events following nuclear delivery of viral genomes. (1) HPV genomes are delivered into the nucleus inside the transport vesicle with a majority of L2 protein's C-terminus on the nuclear side. This event occurs during mitosis. After reformation of the nuclear membrane, L2 protein likely interacts with PML protein through SUMO interactions and recruits PML protein. (2) As the G1 phase continuous to progress, PML protein assembles around HPV-harboring vesicles. (3) Sometime during the engulfment of virus-harboring vesicles by PML protein, the membranous vesicle is lost by an unknown mechanism. We hypothesize that this step allows for the initiation of transcription. (4) Even later in interphase, Sp100 is recruited to the viral genome. The current working hypothesis is that Sp100 recruitment results in repression of viral transcription.

Using our differential staining method and high-resolution imaging, we recently showed that PML protein is recruited to viral genomes and assemble around them, rather than viral genomes targeting preformed PML NBs (Guion et al., 2019) (Figure 1). We also demonstrated that this recruitment occurs in early interphase, soon after completion of mitosis, when PML protein is translocating back to the nucleus. More importantly, PML protein co-localizes with viral genomes that are still residing within transport vesicles. These recent results provided further evidence that PML protein may provide a protective environment for the viral genome in the nucleus of host cells to prevent innate immune recognition. Our data suggest that viral genomes are continuously protected from cellular sensors, first by the transport vesicle, then by PML protein (Figure 2). It seemed also plausible that HPV usurps the dissociation of PML NB during mitosis to modify the composition of PML NB rather than reorganizing preassembled structures.

## SP100 Restricts HPV Transcription

To address this, we extended our studies to the recruitment of Sp100 to PML NBs in HPV infection. Sp100 has been observed to restrict HPV transcription and replication (Stepp et al., 2013, 2017). Indeed, viral transcription and replication were greatly increased in the absence of Sp100 after transfection of HPV 18 genome in primary human keratinocytes (Stepp et al., 2013). Interestingly, the McBride group showed that Sp100 does not seem to be involved in the maintenance phase of the HPV life cycle (Stepp et al., 2017). However, they also showed that Sp100 represses viral processes in later stages as well, during the differentiation-induced viral amplification. In contrast, knockdown of another component of PML NBs, Daxx, did not alter HPV18 transcription and replication suggesting it may not be directly involved in regulating early events of the HPV life cycle. This is in stark contrast to HCMV, where the targeting of DAXX by the tegument protein pp71 is essential for establishing infection (Saffert and Kalejta, 2006).

In our recent findings, we observed a delay in Sp100 recruitment compared to PML protein (Guion et al., 2019). While PML protein was found to be recruited to viral genomes in early interphase, Sp100 was only found co-localizing with viral genomes in late interphase. Furthermore, Sp100 was only observed to co-localize with viral genomes after the loss of the transport vesicles and only when PML protein had already been recruited. Our data suggest that Sp100 is recruited after PML protein. We propose that the delayed recruitment of Sp100 allows an initial burst of viral transcription in absence of Sp100. We furthermore hypothesize that the recruitment of Sp100 may subsequently repress viral transcription thus mediating the switch to the maintenance phase (Figure 2). Interestingly, this was uniquely observed in the presence of viral genomes, suggesting that the recruitment of Sp100 may be directly or indirectly mediated by the virus. So far, the fate of DAXX during these early events of HPV infection has not been evaluated. However, given the fact it does not alter the transcriptional program of HPV18 (Stepp et al., 2013), DAXX may not be affected by incoming HPV virions.

## L2 Protein Interacts With PML NBs

HPV L2 protein is most likely the factor interacting and recruiting PML NB proteins. Although the mechanism is still unknown, it is thought to occur through SUMO interactions. Indeed, one SUMO conjugation motif, one highly conserved SIM and two additional putative SIMs have been identified in the L2 protein sequence (Marusic et al., 2010; Bund et al., 2014). The SUMO conjugation motif was identified at K35 and was shown to play a role in viral capsid assembly (Marusic et al., 2010). However, this motif was not tested for a possible role in interacting with PML protein. The SIMs were identified on the C-terminus of L2 protein at residues 105–109, 145–148, and 286–289 (Bund et al., 2014). The SIM 286–289 was shown to be highly conserved among HPV types and to possibly play a role in interacting with PML protein, but the other SIMs were not tested for such a role.

Our recent findings suggest that L2 protein is also found at PML NBs but remains with the viral genome after loss of the transport vesicle in late interphase (Guion et al., 2019). Similarly to PML protein, SUMO-1 is recruited to and assembles around viral genomes that are still residing within transport vesicles. We also wanted to determine whether the previously identified SUMO conjugation motif and SIMs were involved in recruiting PML protein (Marusic et al., 2010; Bund et al., 2014). However, all mutations in L2 protein caused the PsVs to be impaired for nuclear delivery, most likely interfering with the NRS, similarly to the R302/5A mutant.

## Concluding Remarks

Cells have evolved numerous processes to detect invading pathogens and render their cytoplasm inhospitable. The journey of DNA viruses is particularly treacherous, as cells can detect foreign DNA in the cytoplasm and nucleus and induce an antiviral response (Chan and Gack, 2016). The goal of the antiviral response is to generate an intracellular environment that is both antiproliferative and immunomodulatory in order to restrict viral replication and/or induce the degradation of viral genomes or viral particles (Grandvaux et al., 2002). Therefore, DNA viruses have co-evolved mechanisms to either suppress or evade the host immunity. In the nucleus, PML NBs are the most notable nuclear structures known to be involved in innate immunity and to specifically target viral DNA (Scherer and Stamminger, 2016). Hence, most DNA viruses encode an early protein to dissociate PML NBs and target their proteins for degradation. However, HPVs are very small viruses with limited coding information and thus do not have several factors designed to counteract the host cell immune responses. Instead, we and others were able to show that HPV genomes in complex with viral proteins remain hidden in the cytosol and the nucleus (Day et al., 2004, 2019; Bund et al., 2014; DiGiuseppe et al., 2016, 2017; Bienkowska-Haba et al., 2017; Guion et al., 2019). Unlike other viruses that dissociate PML NBs, HPV takes advantage of their disassembly during mitosis and utilizes them as a new protection device to finally establish infection. Considering our recent findings and other groups' work, we speculate that HPV also utilizes the normal recruitment of Sp100 to PML protein to start its genome maintenance phase. It will be interesting to test if not only the recruitment of Sp100 but also individual PML isoforms are modulated by the presence of HPV, which would support our notion that HPV usurps the disassembly of PML NBs during mitosis to reassemble modified structures around incoming HPV genome. However, additional future studies preferably including a diverse group of researchers are required to confirm these findings.

## Author Contributions

MS and LG contributed to the writing of this review.

### Conflict of Interest

The authors declare that the research was conducted in the absence of any commercial or financial relationships that could be construed as a potential conflict of interest.
